# Daily diet adjustments based on predefined group feeding curves: a practical and scalable approach to enhance nutrient efficiency and sustainability in pig production

**DOI:** 10.3389/fvets.2026.1761763

**Published:** 2026-06-08

**Authors:** Nathalia de Oliveira Telesca Camargo, Julio César Vieira Furtado, Camila Lopes Carvalho, Bruna Souza de Lima Cony, Luciano Hauschild, Luan dos Santos, Raquel Lunedo, Dani Perondi, Ines Andretta

**Affiliations:** 1 Department of Animal Science, Universidade Federal do Rio Grande do Sul, Porto Alegre, Rio Grande do Sul, Brazil; 2 Department of Animal Science, Universidade Estadual Paulista ‘Júlio de Mesquita Filho’, São Paulo, Brazil; 3 Faculty of Veterinary Medicine and Animal Science, Universidade Federal de Mato Grosso do Sul, Campo Grande, Mato Grosso do Sul, Brazil; 4 Master Agroindustrial, Videira, Santa Catarina, Brazil

**Keywords:** amino acids, environmental impact, lysine efficiency, precision feeding, swine

## Abstract

The objective of this study was to evaluate the impact of a scalable feeding strategy based on daily diet adjustments to predefined group feeding curves for growing-finishing pigs, on growth performance, nutrient efficiency, and environmental sustainability responses. Six hundred thirty 64-day-old male pigs were allocated to 30 pens (21 animals per pen), which were randomly assigned to the following treatments: control (conventional phase-feeding program) or daily adjusted diet (same diets but provided in predefined mixing ratios adjusted daily for a gradual transition between feeding phases). Growth performance and nutrient efficiency were assessed using body weight and feed intake records. Serum biochemical responses were analyzed in a representative subsample of the population. Environmental impact was inferred based on estimates of the global warming, terrestrial acidification, freshwater eutrophication, and land use associated with feeding practices. All data were tested using analysis of variance, and the results were interpreted at a significance level of 5%. No differences were observed in growth performance or backfat thickness between the treatments. However, daily adjusted feeding program allowed a 5% reduction in standardized ileal digestible (SID) lysine intake (*p* < 0.05), consequently improving SID lysine efficiency by 6% (*p* < 0.05). Serum urea concentrations were consistently lower in the daily adjusted feeding program (*p* < 0.05), indicating more efficient protein utilization. Daily adjusted feeding program also mitigated (*p* < 0.05) the potential impacts of global warming (up to 6%), acidification (up to 3.5%), eutrophication (up to 9%), and land use (up to 3%). These findings demonstrate that a scalable precision feeding strategy can improve nutrient efficiency and metabolic responses without compromising growth performance, representing a practical solution for enhancing sustainability in commercial pig production systems.

## 1 Introduction

A key challenge faced by the pig sector is to produce high-quality protein at the lowest possible cost (1). Addressing this challenge is not a simple task, and it requires the adoption of innovative approaches. As the sector continues to expand, strategies such as precision feeding are emerging as promising alternatives for enhancing both production efficiency and sustainability (2). This approach has gained attention for its potential to reduce feeding costs while providing animals with exactly the nutrients needed (1). This relevance is further emphasized by the fact that feeding accounts not only for a significant portion of production costs, but also for the largest share of environmental impacts in pig systems (1).

Conventional phase-feeding protocols employ a restricted sequence of static dietary formulations (typically 3 to 6 phases) that are uniformly delivered to all animals within production groups throughout predetermined growth intervals (1–3). Although widely adopted, such programs do not account for the fact that nutritional requirements vary among animals and evolve dynamically throughout growth (4), leading to reduced nutrient-use efficiency, as animals often receive nutrients in excess of their actual needs (1). Variation among animals at a given age reflects differences in body weight, growth potential, efficiency of nutrient utilization, or growth composition (2, 5, 6). To address this variability, precision feeding strategies incorporating daily or individual adjustments have been explored. Notably, Andretta et al. (7) demonstrated that precision feeding techniques can reduce protein intake by up to 25% and nitrogen excretion by up to 40% while maintaining growth performance comparable to conventional feeding programs. In addition, individual precision feeding has been shown to reduce environmental impacts across multiple categories, including climate change, energy demand, eutrophication, acidification, and land use (7–9).

Despite these promising results, the implementation of individual adjusted feeding programs in the daily operation of commercial farms remains challenging (10, 11). Feeding pigs individually requires specialized equipment, which may be costly and have limited availability in the market (12). In addition, these benefits are often related to the use of mathematical models adapted to operate in real-time, requiring sensors that are not available on most commercial farms (10).

A more immediate and practical approach for improving the precision of feeding strategies under field conditions would be to apply daily diet adjustments based on predefined (rather than updated in real-time) feeding curves tailored to the group (rather than the individual) (13). This type of adjustment could already be implemented in production systems equipped with dry or wet automated feeding systems or automated feeding robots (14). Even though less precise than the individual approach, this technically feasible solution represents a practical step toward broader adoption, offering immediate applicability, while more complex systems continue to evolve. The objective of this study was to evaluate the impact of a scalable daily adjusted feeding program based on predefined feeding curves and daily diet adjustments for a group of growing-finishing pigs on growth performance, nutrient efficiency, and environmental sustainability.

## 2 Materials and methods

### 2.1 Animals, housing conditions, and experimental treatments

All procedures were carried out with the approval of the Ethics Committee on Animal Use of the Universidade Federal do Rio Grande do Sul (Porto Alegre, Brazil). This study was conducted in a commercial production facility located in Videira, Santa Catarina, Brazil. A total of 630 male pigs (Agroceres PIC, São Paulo, Brazil) were housed in a barn with 30 pens of 18 m^2^ with partially slatted floor.

The room was naturally ventilated using adjustable side-curtains. Temperature and humidity were recorded continuously using data loggers distributed in the barn. Air quality was monitored throughout the experimental period, whereas ammonia concentrations were registered weekly in each pen using a gas detector device (KR1320 Ammonia Gas Detector, Akrom, São Paulo, Brazil). Water was provided *ad libitum* through low-pressure nipple drinkers, and feed was offered four times a day in linear feeders (30–35 cm per animal). Standard management practices of the production unit were maintained throughout the trial. For instance, the animals began the project as intact males and were immunocastrated using two doses of a vaccine (Vivax, Zoetis, São Paulo, Brazil) applied on days 37 and 50 of the experimental period. The trial lasted 105 days.

Animals were individually weighed during housing and distributed among pens to maintain similar average body weights across pens at the start of the experimental period. All animals received the same commercial diet for 16 days, which served as the pre-experimental phase. The phase was conducted, not only as an adaptation period for the animals, but also to verify and stabilize the operational accuracy of the robotic feeding system, particularly regarding feed blending and delivery precision.

After the adaptation period, the experimental treatments were implemented (mean body weight: 35.79 kg; standard deviation: 4.77 kg). Pens were randomly allocated to either a control or daily adjusted feeding treatment. The control group received a conventional phase-feeding regimen consisting of four fixed dietary phases: phase 1 (days 1–28), phase 2 (days 29–49), phase 3 (days 50–61), and phase 4 (days 62–89), with all animals within each phase receiving identical feed formulations. In contrast, the daily adjusted feeding program employed the same dietary formulations but implemented gradual daily transitions between phases through predetermined proportional mixing of adjacent phase diets (13), maintaining consistent blending ratios throughout the experimental period. The daily adjusted feeding strategy was applied at the treatment level (barn-level curve), meaning that all pens assigned to the adjusted program received the same predefined daily dietary blending ratios rather than pen-specific adjustments. No adjustments were made to the dilution proportions or feeding curves during the trial period.

A phase-feeding program was adopted in the control treatment as it represented the farm’s existing management strategy. Diet formulations were based on the models described in the Brazilian Tables for Poultry and Swine (15) and adjusted according to estimated feed intake curves derived from previous batches raised under similar commercial conditions on the same farm. Additionally, a fifth diet was formulated based on the estimated nutrient requirements for the final phase of the trial (16), allowing for the dilution of the fourth-phase feed in the daily adjusted feeding program. Feeds were formulated using the least-cost procedures and included ingredients that are commonly used in the region (Table 1). No additional safety margin was applied during diet formulation. As in typical phase-feeding systems, this approach may result in nutrient supply exceeding the requirements of some animals during part of the phase due to the natural decline in requirements as pigs grow. Feed samples were collected weekly, and one composite sample per feeding phase was analyzed for crude protein, gross energy, calcium, and phosphorus, confirming values close to those targeted during the formulation. All feeds were offered in mashed form.

**Table 1:** Nutritional composition of experimental diets across swine growth phases (as-fed basis).

	Growth I	Growth II	Finishing I	Finishing II	Finishing III
Ingredients, %					
Corn (8% CP^1^)	72.31	77.49	74.78	77.29	83.34
Soybean meal (46% CP)	20.38	16.04	9.68	8.04	8.18
Meat meal (50% CP)	3.50	3.50	3.00	2.25	1.37
DDGS^2^ (42% CP)	—	—	10.00	10.00	4.93
Soybean oil	1.69	1.00	0.69	0.32	—
L-Lysine HCl (54%)	0.65	0.65	0.65	0.65	0.56
Salt	0.46	0.43	0.42	0.44	0.46
Limestone	0.38	0.28	0.36	0.60	0.77
Vitamin-mineral premix*	0.64	0.62	0.42	0.43	0.39
Analyzed composition, %					
Dry matter	87.72	87.58	87.52	87.45	87.33
Crude protein	17.66	16.09	16.79	15.83	13.66
Crude fiber	2.26	2.13	1.78	1.74	1.85
Calculated composition, %					
Metabol. energy (kcal/kg)	3,405	3,383	3,370	3,352	3,339
Net energy (kcal/kg)	2,550	2,550	2,556	2,550	2,550
SID amino acids, %					
Lysine	1.10	1.00	0.90	0.85	0.75
Methionine + Cysteine	0.63	0.57	0.52	0.50	0.44
Threonine	0.68	0.63	0.59	0.58	0.51
Tryptophan	0.21	0.19	0.17	0.16	0.14
Minerals, %					
Calcium	0.65	0.60	0.58	0.60	0.60
Total phosphorus	0.42	0.41	0.42	0.39	0.33
Available phosphorus	0.37	0.37	0.34	0.31	0.28

*The premix contained the following quantities of vitamins, amino acids, and minerals per kilogram of diet: 12,000 IU vitamin A, 2,000 IU vitamin D₃, 100 mg vitamin E, 3 mg vitamin K₃, 2 mg vitamin B₁, 8 mg vitamin B₂, 5 mg vitamin B₆, 25 mg calcium-D-pantothenate, 0.04 mg vitamin B₁₂, 30 mg niacin, 550 mg choline, 4 mg folic acid, 0.50 mg biotin, 150 mg iron, 15 mg copper, 50 mg manganese, 70 mg zinc, 2 mg iodine, 0.4 mg selenium, 18.3 mg lysine, 9.80 mg methionine, 18.3 mg threonine, and 7.89 mg tryptophan.

^1^CP: crude protein.

^2^DDGS: distillers dried grains with solubles.

The feed was delivered automatically using a robotic device (Roboagro, Caxias do Sul, Brazil) designed for commercial pig production. This robot moved along the central corridor of the barn and dispensed feed directly into the linear feeders in each pen. The device was equipped with two separate compartments, each of which could be loaded with a different type of feed, allowing customized blends to be prepared according to the treatment designation of each pen. The system was programmed to provide four daily feedings based on predefined nutrient curves for each treatment.

The feed supply was controlled and updated weekly according to an estimated curve based on previous batches raised under similar conditions on the same farm (16) along with the recommendations of the Brazilian Tables for Poultry and Swine (15). This approach was adopted to ensure that feed delivery closely matched the expected consumption pattern of the animals, thereby minimizing potential negative effects associated with scheduled feeding, such as excessive competition at feeding times, unequal feed access among pen mates, frustration, and abrupt changes in feeding behavior. At each phase change, the equipment cycle was manually reprogrammed to provide the necessary amount of feed to the animals according to the experimental protocol. Feed delivery precision was monitored daily by collecting and weighing the feed dispensed into an additional pen designated specifically for this purpose.

### 2.2 Data collection

The animals were weighed individually at the beginning and end of the project, while pen-level weighing was performed at each transition between the feeding phases. Backfat thickness was measured on the same days as body weight assessments using a portable ultrasound device (Renco Lean Meater, MS-Schippers, São Paulo, Brazil) equipped with a 3.5 MHz linear transducer. Measurements were performed on three pigs per pen, selected based on their initial body weights to represent the lightest, medium, and heaviest animals within each pen. The measurement site was the P2 position, located approximately 6.5 cm laterally from the dorsal midline at the level of the last rib. Pigs were kept standing during the procedure, and the transducer was placed directly on the skin after applying coupling gel. Three consecutive readings were taken per animal, and the average of these values was used as the final backfat thickness. This methodology provided a precise and non-invasive evaluation of subcutaneous fat deposition throughout the experimental period. The health status of the pigs was monitored daily.

Feed supply was recorded using the automated feeding system at the pen level, and feed intake was calculated by subtracting any feed refusals (weighed daily in each pen). Feed efficiency (gain-to-feed ratio) was estimated for each period and adjusted for mortality (accounting for the body weight of animals that died or were removed during the experimental period in the estimation of total pen weight gain). Standardized ileal digestible (SID) lysine intake was obtained based on the calculated feed composition. Nutrient-use efficiency was obtained by considering animal growth and assuming similar rates of lysine deposition in weight gain across treatments and throughout the trial (7). Therefore, this index was interpreted as a calculated indicator of lysine use relative to growth performance, rather than as a direct measurement of lysine deposition or retention.

At the end of each feeding phase, blood samples were collected from one animal per pen (with medium body weight at the beginning of the trial). Thus, serum biochemical responses were analyzed using the pen as the experimental unit (based on a single representative animal per pen). Consequently, within-pen variation was not captured in these responses. Sampling was conducted prior to routine handling procedures on the designated sampling day, and an 8–10 h fasting period was implemented before blood collection. Blood was collected in tubes without anticoagulants and centrifuged at 3500 rpm for 10 min. The resulting serum was carefully collected and frozen at −20 °C. Serum concentrations of total protein and urea were analyzed at all sampling points, whereas albumin was measured only at the beginning and at the end of the trial. Glucose, cholesterol, triglycerides, creatinine, alanine aminotransferase (ALT), aspartate aminotransferase (AST), and alkaline phosphatase (ALP) were measured only at the end of the trial. Analyses were performed using commercial kits (Wiener Lab Group, São Paulo, Brazil) and a biochemical analyzer (Bio-Plus 2000®, Bioplus, São Paulo, Brazil).

### 2.3 Environmental impacts

Potential environmental impacts associated with feeding practices were estimated based on the ingredient composition of each feed formulation using a functional unit of 1 ton of feed. Global warming, terrestrial acidification, freshwater eutrophication, and land-use categories were considered. Impact data were obtained from the Global Feed LCA Institute (GFLI) datasets (Arlington, Virginia, USA). Two environmental impact scenarios were analyzed, differing in the impact values used to characterize feed ingredients. In the first scenario, the GFLI database was applied to all ingredients. In the second scenario, the same database was used for all ingredients except soybean meal, for which updated impact values provided by Embrapa were incorporated. Locally adjusted emission factors were applied only to soybean meal because the main nutritional change promoted by the feeding strategy was associated with protein supply rather than energy supply.

The potential environmental impact of feeding was calculated by multiplying the impact per ton of feed by the feed intake obtained in the experiment. These values were then normalized by the corresponding weight gain, yielding results expressed as environmental impact per kilogram of pigs produced. All estimates were calculated on a pen-level basis.

### 2.4 Statistical analyses

Data analyses were performed using SAS statistical program (v. 9.3, SAS Institute Inc., Cary, North Carolina, USA), with the pen as the experimental unit. The data were subjected to analysis of variance using PROC MIXED. All the statistical models included the fixed effects of treatment. For responses measured repeatedly over time (i.e., performance), the model included the fixed effects of treatment, phase, and the treatment × phase interaction. Pen nested within treatment was used as the subject identifier for the repeated statement. The covariance structure was selected based on the lowest Akaike information criterion among the tested structures. Residual normality was tested using the Shapiro–Wilk test with PROC UNIVARIATE. Finally, the results were interpreted at 5 and 10% significance levels.

## 3 Results

### 3.1 General conditions

The minimum and maximum temperatures registered during the trial were 18 and 29 °C, respectively, while the relative humidity ranged from 72 to 79%. These environmental conditions are considered adequate for growing-finishing pigs. The ammonia levels varied from 0 to 2 ppm; therefore, remaining within the acceptable limits for pigs. The animals exhibited performance responses consistent with expectations for their specific genotypes throughout the entire trial. No relevant health issues, signs of cannibalism, skin lesions, or other significant injuries were observed throughout the experiment. Minor locomotor problems were identified in 14 animals. These animals were isolated in separate pens to avoid worsening of their condition and were later removed from the analysis.

### 3.2 Performance and nutrient-use efficiency

**Figure 1:**
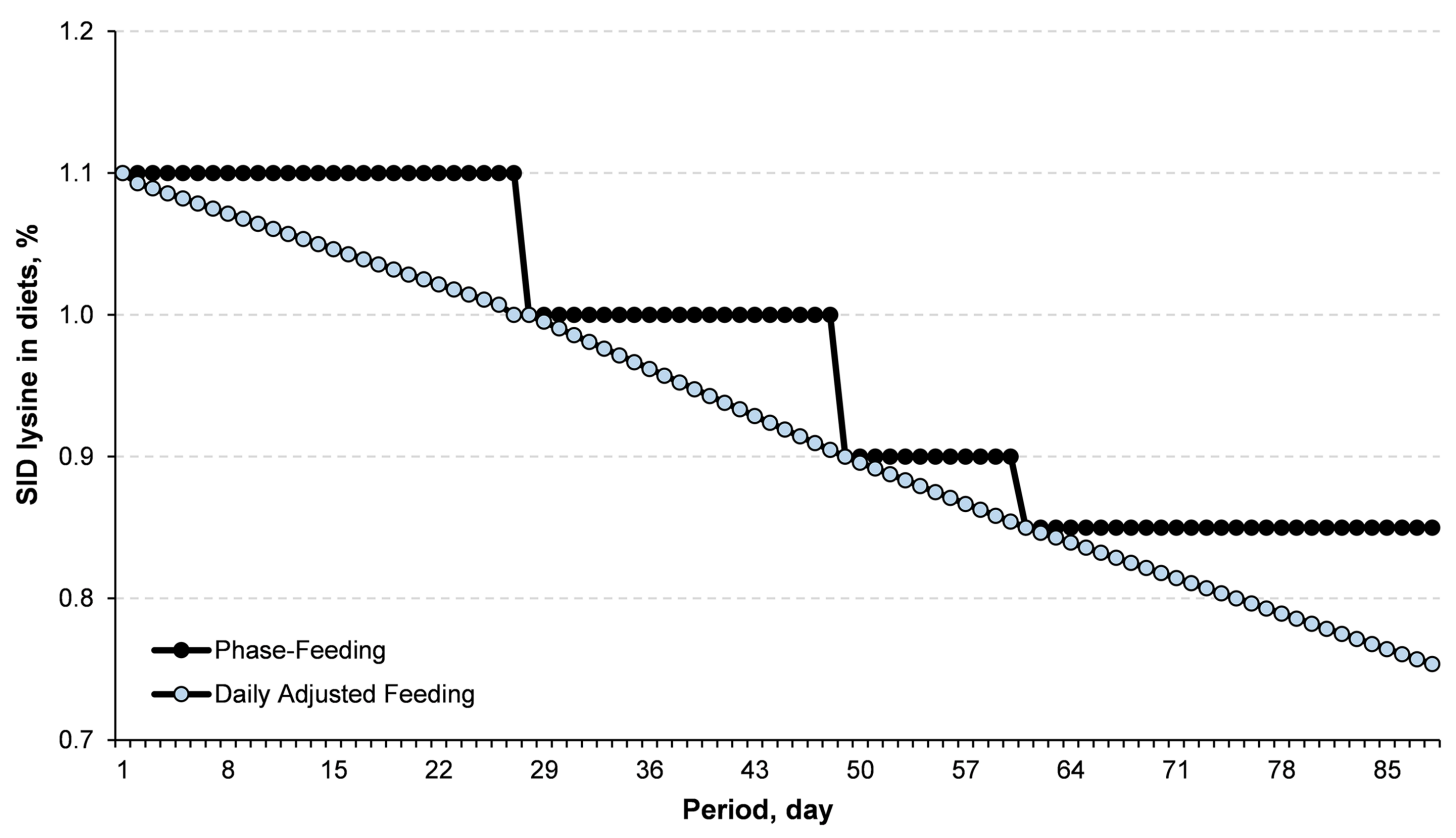
Standardized ileal digestible (SID) lysine levels in diets for pigs reared under conventional phase-feeding or daily adjusted feeding programs.

**Figure 2:**
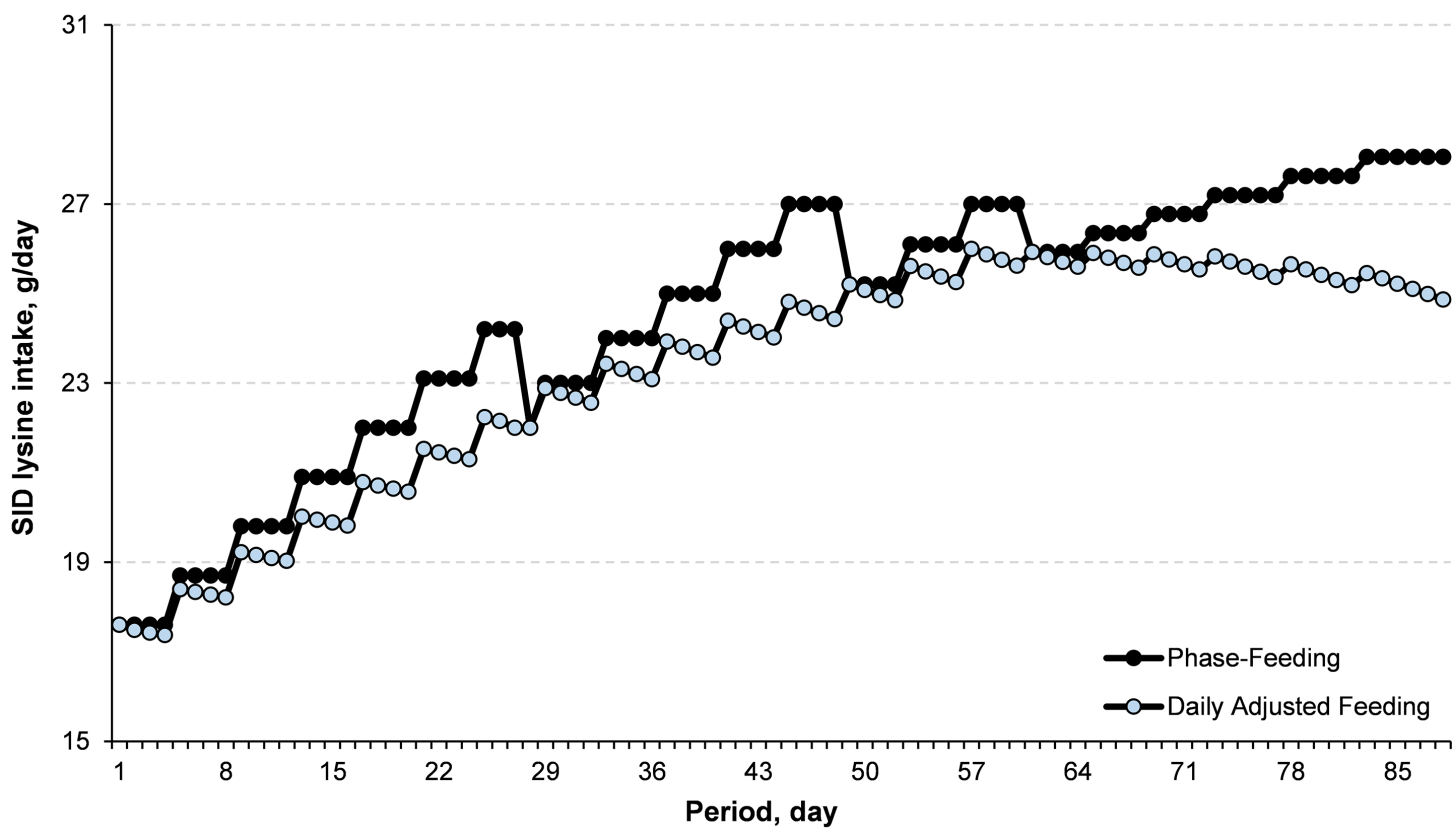
Daily standardized ileal digestible (SID) lysine intake in pigs reared under conventional phase-feeding or daily adjusted feeding programs.

As anticipated, both the dietary concentrations (Figure 1) and daily intake of SID lysine (Figure 2) were consistently lower in the daily adjusted feeding program than in the control group throughout the trial. The implementation of the daily adjusted strategy resulted in a 4.7% reduction in dietary SID lysine concentration. Consequently, pigs managed under the daily adjusted feeding program showed a 4.9% decrease in total SID lysine intake compared with those receiving the conventional phase-feeding program.

**Figure 3:**
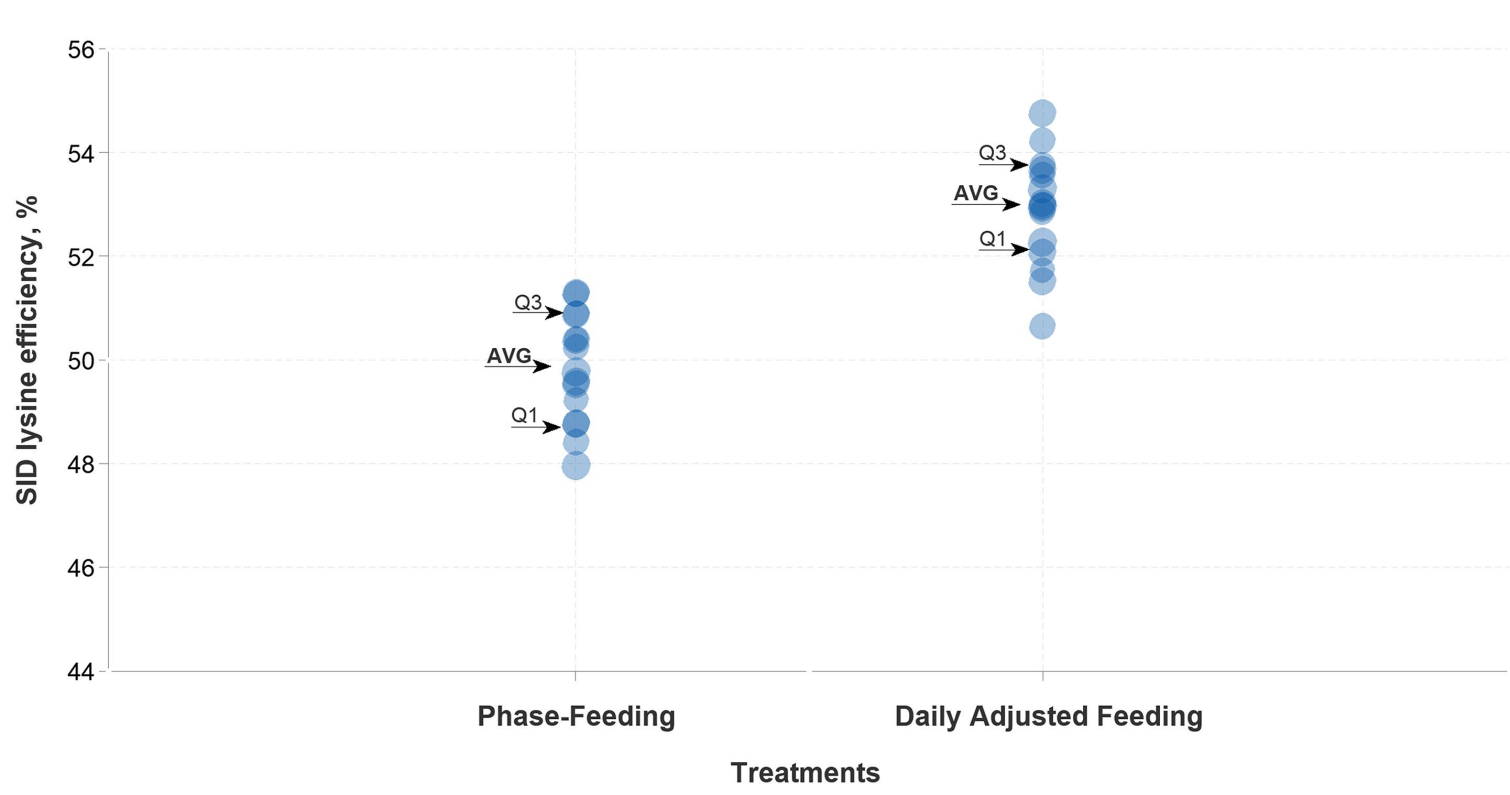
Standardized ileal digestible (SID) lysine efficiency relative to weight gain in pigs reared under conventional phase-feeding or daily adjusted feeding programs. Q1, first quartile; AVG, average value; Q3, third quartile. The circle size varies with the mean backfat thickness in each pen.

The treatments resulted in similar body weights and backfat thicknesses throughout the trial (Table 2). The coefficient of variation was also unaffected by the treatment. Similarly, feed intake, weight gain, and feed efficiency were comparable between treatments in all feeding phases, as well as in the overall means (Table 3). However, SID lysine efficiency was improved (*p* < 0.05) by the daily adjusted feeding program across all phases and in the total period. On average, animals raised under daily adjusted program showed SID lysine efficiency 6% higher than that of the control group (*p* < 0.05; Figure 3), with a 12.5% improvement during the final phase of the trial.

**Table 2:** Body weight (BW) and backfat thickness (BT) of growing pigs reared under conventional phase-feeding or daily adjusted (DA) feeding programs.

	Treatments		RSE^1^	*p-*value
	Control	DA		
Day 1				
BW, kg	35.74	35.84	0.30	0.878
CV of BW^2^, %	12.60	12.87	0.36	0.715
BT, mm	4.266	4.212	0.02	0.280
Day 28 (end of phase 1)				
BW, kg	63.24	63.61	0.42	0.676
BT, mm	4.371	4.400	0.03	0.774
Day 49 (end of phase 2)				
BW, kg	87.09	87.47	0.47	0.701
BT, mm	6.644	6.490	0.07	0.267
Day 61 (end of phase 3)				
BW, kg	101.1	101.7	0.48	0.550
BT, mm	7.195	7.499	0.10	0.156
Day 89 (end of phase 4)				
BW, kg	130.6	132.6	0.72	0.174
CV of BW^2^, %	9.330	9.782	0.29	0.449
BT, mm	9.928	9.893	0.14	0.915

^1^RSE: residual standard error.

^2^CV of BW: coefficient of variation of body weight, calculated within each pen.

**Table 3:** Growth performance of growing pigs reared under conventional phase-feeding or daily adjusted (DA) feeding programs.

	Treatments		RSE^1^	*p-*value
	Control	DA		
Day 1 to 28 (phase 1)				
ADFI^2^, g/day	1,845	1,841	9.31	0.810
ADG^3^, g/day	982.0	992.7	10.1	0.613
G:F^4^, g/g	0.521	0.526	0.01	0.684
SID^5^ lysine efficiency, %	53.27	56.33	0.56	0.012
Day 29 to 49 (phase 2)				
ADFI^2^, g/day	2,456	2,456	9.34	0.973
ADG^3^, g/day	1,136	1,136	11.3	0.926
G:F^4^, g/g	0.453	0.452	0.01	0.874
SID^5^ lysine efficiency, %	50.87	53.54	0.51	0.009
Day 50 to 61 (phase 3)				
ADFI^2^, g/day	2,908	2,896	4.32	0.297
ADG^3^, g/day	1,402	1,424	16.0	0.477
G:F^4^, g/g	0.483	0.491	0.01	0.488
SID^5^ lysine efficiency, %	60.23	64.70	0.67	0.029
Day 62 to 89 (phase 4)				
ADFI^2^, g/day	3,313	3,329	5.24	0.201
ADG^3^, g/day	1,040	1,057	9.34	0.219
G:F^4^, g/g	0.335	0.352	0.01	0.214
SID^5^ lysine efficiency, %	40.01	45.41	0.54	0.012
Day 1 to 89 (overall period)				
ADFI^2^, g/day	2,603	2,606	6.21	0.820
ADG^3^, g/day	1,085	1,097	4.14	0.117
G:F^4^, g/g	0.414	0.423	0.01	0.104
SID^5^ lysine efficiency, %	49.80	52.91	0.33	<0.001

^1^RSE: residual standard error.

^2^ADFI: average daily feed intake.

^3^ADG: average daily weight gain.

^4^G:F: feed efficiency.

^5^SID: standardized ileal digestible.

The serum concentrations of total protein, albumin, creatinine, ALT, AST, ALP, cholesterol, triglycerides, and glucose did not differ between the treatments (Table 4). However, daily adjusted program reduced (*p* < 0.05) serum urea concentration, and this reduction ranged from a minimum of 10% to a maximum of 35% at the end of the first and second phases, respectively.

**Table 4:** Serum biochemical responses in growing pigs reared under conventional phase-feeding (control) or daily adjusted (DA) feeding programs.

	Treatments		RSE^1^	*p-*value
	Control	DA		
Day 28 (end of phase 1)				
Total protein, mg/dL	5.260	5.246	0.62	0.954
Urea, mg/dL	37.36	33.60	9.16	0.279
Albumin, mg/dL	4.129	4.053	0.48	0.678
Day 49 (end of phase 2)				
Total protein, mg/dL	5.167	5.173	0.49	0.971
Urea, mg/dL	31.20	20.36	7.00	<0.001
Day 61 (end of phase 3)				
Total protein, mg/dL	4.460	4.713	0.48	0.163
Urea, mg/dL	44.40	31.93	8.23	<0.001
Day 89 (end of phase 4)				
Total protein, mg/dL	7.827	8.407	1.56	0.319
Urea, mg/dL	63.20	50.36	12.9	0.012
Albumin, mg/dL	4.633	4.753	0.67	0.626
Creatinine, mg/dL	3.860	3.713	0.54	0.464
ALT^2^, U/L	31.40	31.53	7.03	0.959
AST^3^, U/L	15.60	14.73	2.86	0.413
Alkaline phosphatase, U/L	465.3	446.1	77.4	0.502
Cholesterol, mg/dL	98.19	86.31	21.9	0.148
Triglycerides, mg/dL	79.87	87.73	17.1	0.219
Glucose, mg/dL	165.8	172.5	33.4	0.585

^1^RSE: residual standard error.

^2^ALT: alanine aminotransferase.

^3^AST: aspartate aminotransferase.

### 3.3 Environmental impacts

**Figure 4:**
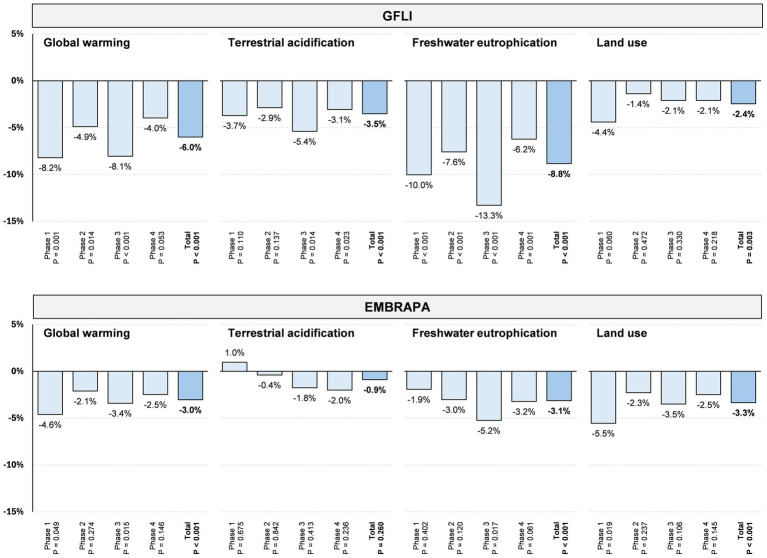
Change (%) attributed to daily adjusted feeding program on the potential environmental impacts of feeding growing-finishing pigs. Impacts were estimated considering the feed amount consumed by each pen in the trial and the relative impact attributed to each ingredient in the feed formula. Later, the feeding impacts were adjusted (divided) by the weight gain of each pen, resulting in values expressed as the impact (kg CO_2_eq (global warming)/kg SO_2_eq (terrestrial acidification)/kg Peq (freshwater eutrophication)/m^2^a (land use)) attributed to the feed necessary to produce 1 kg of live weight gain (functional unit). The difference (%) between control and daily adjusted feeding programs is presented in the figure. Probability of treatment effect is indicated for each response.

The potential environmental impacts associated with feeding practices were consistently reduced by the implementation of daily adjusted feeding program (Figure 4). The greatest reductions were observed when the simulations were fully based on data from the GFLI database. In this scenario, the global warming potential was reduced by an average of 6% (*p* < 0.05), with the greatest reduction observed in phases 1 and 3 (−8%). Terrestrial acidification and freshwater eutrophication potentials were also reduced by 3.5% (*p* < 0.05) and 9% (*p* < 0.05), respectively, with phase-specific reductions reaching up to −13% for eutrophication in phase 3. Land use was mitigated by 2% over the entire study period (*p* < 0.05), with the largest decrease reported in phase 1 (−4%). The mitigation associated with daily adjusted feeding program was also observed for some categories when the EMBRAPA-adjusted database was used to incorporate region-specific values for soybean meal into simulations. Although the effects observed in this scenario were generally less pronounced, daily adjusted feeding program still mitigated the potential impacts of global warming, eutrophication, and land use by 3% (*p* < 0.05).

## 4 Discussion

Conventional feeding systems are generally based on fixed feed formulas provided to groups of animals over specific periods/phases. Although widely adopted in pig production worldwide, the phase-feeding programs do not fully account for the variability in nutrient requirements among individuals and throughout their growth (12). In contrast, adjusted feeding programs have been proposed to improve the adjustment between the nutrient supply in the diets and the nutritional requirements of animals (2). Advanced technologies and mathematical modeling are often employed to achieve this goal (9). However, it is important to highlight that precision feeding is a broad concept that may encompass strategies with varying levels of complexity (14). A thorough understanding of the nutritional requirements of animals is one of the key criteria for proposing effective adjusted feeding programs (2). Since nutritional requirements change dynamically throughout the growth of an animal, implementing frequent (e.g., daily) dietary adjustments is often considered a critical step toward more precise feeding programs (9). The complexity of such systems can be increased, as these adjustments can be applied at either the individual or group level (8). In addition, the modifications may be based on historical performance data from similar populations or guided by real-time monitoring systems (16).

In this study, precision feeding was implemented using predefined nutritional curves to adjust the diets daily at the group level. Although this approach may not represent the highest degree of precision, compared to individualized real-time systems, it still offers important advantages. First, this strategy is probably one of the most feasible for immediate adoption on commercial farms because it depends on infrastructure and management tools that are already available in many production systems (12). In addition, although previous studies have demonstrated the benefits of feeding pigs individually, the largest part of these gains can be achieved through daily diet adjustments, even when applied at the group level (2, 17, 18). A previous study compared conventional phase-feeding and daily adjusted feeding program applied to groups or individuals (18). In this study, more than half of the total benefit was already achieved with the daily adjusted feeding program, as observed in relevant responses such as SID lysine intake (17% reduction for group-based vs. 27% for individual-based) and estimated excretion of nitrogen (12% vs. 22%) and phosphorus (15% vs. 27%). A complementary study also showed that while precision feeding models tailored daily to individual pigs resulted in up to 6% reduction in climate change impact, daily group-based precision feeding systems were able to achieve a reduction of up to 4% in the same impact (17).

In the current trial, SID lysine intake was reduced by 5%, while SID lysine efficiency was improved by 6%, compared to the control group. These two responses are intrinsically linked, because SID lysine efficiency was estimated from growth performance relative to SID lysine intake, assuming similar lysine deposition in weight gain between treatments. Thus, the increase in this index should be interpreted primarily as the arithmetic consequence of reducing SID lysine intake while maintaining similar weight gain, rather than as independent evidence of altered lysine retention. The impacts observed in this trial were lower than those reported in previous studies in which protein deposition was measured using DXA technology (8, 18), likely because the current study did not employ real-time adjusted models and also because the control treatment already included a higher level of nutritional refinement (i.e., using four instead of three phases). Nevertheless, the biological and practical relevance of these results should not be underestimated. Reducing nutrient intake while maintaining growth performance indicates that the animals’ requirements were met more closely, with less nutritional oversupply. This interpretation is further supported by the lower serum urea concentrations observed in pigs receiving the daily adjusted feeding program, which provide an independently measured metabolic indication of reduced amino acid catabolism and more efficient nitrogen utilization.

From a production perspective, these results are particularly important because they reflect a more efficient use of nutritional resources without compromising productive responses. In addition, this type of result is highly relevant from an environmental standpoint, as lower nutrient input tends to reduce the burden associated with feed use and the risk of nutrient losses. This gain represents a highly relevant improvement for production systems, especially when considering that animal performance was not affected. It is also important to note that the experimental group in this study was relatively homogeneous in terms of sexual category and age. In commercial systems that house multiple age groups or sexual categories, potential gains from precision feeding may be even greater (16). Systems capable of applying multiple predefined curves in the same barn (e.g., across pens) can produce even greater gains.

The lack of an impact on performance is an outcome consistently reported in multiple studies on precision feeding (8, 18). Pigs also exhibit feeding behaviors similar to those of conventional or adjusted feeding programs. Body composition outcomes were similar between treatments in the current study (backfat thickness) and in previous reports (8, 18). However, low-protein diets may promote lipogenesis under certain conditions (19, 20), underscoring the importance of precisely balancing amino acid supply when formulating feeds. Notably, pigs managed under precision feeding had lower serum urea concentrations, corroborating previous findings (8, 15, 21). Importantly, this reduction occurred without changes in serum total protein levels, suggesting a more efficient routing of dietary nitrogen toward protein accretion rather than excretion. Serum biochemical analyses were performed using one representative pig per pen, which means that within-pen variation was not captured. Therefore, although the reduction in serum urea was consistent and biologically coherent with the lower SID lysine intake, this response should be interpreted as a pen-level indication based on representative sampling rather than a full characterization of individual variability within pens.

Because this trial was conducted under commercial field conditions, no direct assessment of whole-body composition or nutrient retention was performed, which prevents a robust estimation of nitrogen and phosphorus deposition in the animals. Although lysine-use efficiency was calculated, this index was based on the assumption that tissue deposition was not altered by the treatments, an aspect that cannot be fully confirmed based only on growth performance and backfat thickness measurements. However, it is notable that precision feeding offers substantial environmental benefits, mainly by reducing nutrient excretion and improving the use efficiency of high-impact resources (e.g., soybean meal and inorganic phosphorus). Previous studies have reported reductions of up to 22% in climate change impact and 13% in eutrophication potential when feeding systems were adjusted in real time at the individual level (10, 11, 17). In the current study, using predefined curves and daily adjustments at the group level, the global warming potential was reduced by up to 6 percent, demonstrating that even simplified precision feeding strategies can deliver significant sustainability benefits.

The magnitude of the environmental mitigation observed depended also on the dataset used for estimating feed ingredient impacts, particularly for soybean meal, which is one of the major contributors to the environmental footprint of pig diets. When the GFLI dataset was applied to all ingredients, the estimated benefits of precision feeding were greater. In contrast, when region-specific EMBRAPA values were used for soybean meal, the reductions became less pronounced, indicating that the absolute mitigation potential is sensitive to the impact attributed to this ingredient. Although this sensitivity to the database does not undermine the feeding strategy, it highlights that the estimated environmental benefits are context-dependent. Therefore, extrapolation of these results to other production systems, ingredient sources, or regional conditions should be made with caution.

The daily adjusted feeding program evaluated in this study proved to be both effective and readily applicable under commercial conditions, improving nutrient-use efficiency while reducing environmental impact. Although more advanced systems based on real-time data and individual animal monitoring may further increase biological precision, their implementation in commercial pig production is still constrained by technological, operational, and economic limitations (22, 23). In contrast, the system tested here relied on predefined group-level feeding curves, daily blending of adjacent diets, and a commercially available robotic feeder, without requiring real-time sensor inputs or individual-level decision making. Thus, while this approach does not represent the highest level of precision described in scientific literature, it constitutes a realistic and immediately deployable alternative to conventional phase feeding. Importantly, the present findings demonstrate that a substantial proportion of the benefits associated with precision feeding can already be achieved through simple, scalable, and operationally feasible adjustments in feed delivery. This is particularly relevant because automated feeding robots are often used below their full potential, serving mainly as labor-saving tools in the farms, whereas their strategic use for nutritional management may generate additional technical, economic, and environmental value. Overall, these results support the view that the transition toward more sustainable pig production will depend not only on highly sophisticated technologies, but also on practical solutions that can be adopted in the short term and deliver immediate benefits to both producers and the sustainability of the sector.

## 5 Conclusion

The daily adjusted feeding program effectively enhances the resource-use efficiency of pig production. This approach supports more sustainable production systems by reducing nutrient oversupply, without compromising growth performance. Continued research is nevertheless essential to refine models and support the broader adoption of these strategies across diverse production systems.

## Data Availability

The dataset generated and analyzed in this study is not publicly available due to commercial restrictions from the collaborating company. Additional information may be provided by the authors upon reasonable request and subject to company approval. Requests to access the datasets should be directed to Nathalia Camargo, nathytcamargo@gmail.com.
